# Heart rate-corrected QT interval prolongation is associated with decreased heart rate variability in patients with type 2 diabetes

**DOI:** 10.1097/MD.0000000000031511

**Published:** 2022-11-11

**Authors:** Seon-Ah Cha

**Affiliations:** a Division of Endocrinology and Metabolism, Department of Internal Medicine, St. Vincent’s Hospital, College of Medicine, The Catholic University of Korea, Seoul, Republic of Korea; b Division of Endocrinology and Metabolism, Department of Internal Medicine, Wonkwang University Sanbon Hospital, Gunpo, Republic of Korea.

**Keywords:** heart rate variability, QT interval, type 2 diabetes

## Abstract

We investigated the association between the heart rate-corrected QT interval (QTc interval) measured by standard electrocardiography and heart rate variability (HRV) in patients with type 2 diabetes mellitus (T2DM). From March 1, 2009, to December 12, 2009, 411 patients with T2DM who underwent resting 12-lead electrocardiography and cardiovascular autonomic function testing concurrently without the exclusion criteria were consecutively recruited in this cross-sectional study. Time- and frequency-domain HRV variables were assessed for 5 minutes by beat-to-beat HRV recording. The QT interval was corrected for the heart rate using Bazett’s formula. QTc interval measurements of >440 ms were considered abnormally prolonged. The mean age and diabetes duration were 56.3 ± 10.6 years and 9.6 ± 7.3 years, respectively. A total of 90 patients had QTc interval prolongation (21.9%). The participants with a prolonged QTc interval were older (59.4 ± 10.1 years vs 55.5 ± 10.6 years, *P* = .002), were more likely to be a woman (72.2% vs 51.7%, *P* = .001), had a higher prevalence of hypertension (46.7% vs 33.4%, *P* = .022), had a higher hemoglobin A1c level (8.8% ± 2.2% vs 8.2% ± 1.8%, *P* = .045), and had decreased values for the variables measuring HRV, except for the low frequency (LF)/high frequency (HF) ratio (total power [TP], 147.7 [74.1–335.9] ms vs 328.7 [185.7–721.7] ms, *P* = .002). After adjusting for multiple confounders, QTc interval prolongation was associated with the lowest quartile of the HRV parameters of TP (odds ratio [OR] = 3.99; 95% confidence interval [CI]: 2.29–6.96), HF (OR = 3.20; 95% CI: 1.84–5.58), LF (OR = 3.68; 95% CI: 2.10–6.43), standard deviation of the normal-to-normal interval (OR = 3.31; 95% CI: 1.89–5.77), and root-mean-square of the successive differences (OR = 1.98; 95% CI: 1.13–3.47) in patients with T2DM. Decreased values for the variables measuring HRV, except for the LF/HF ratio, might be associated with QTc interval prolongation in patients with T2DM.

## 1. Introduction

Type 2 diabetes mellitus (T2DM) affects 10.5% of the worldwide adult population aged 20 to 79 years, and this percentage is estimated to increase to 12.2% by 2045.^[1]^ Previous cohort studies have demonstrated that those with T2DM or type 1 DM (T1DM) have a 1.25-fold-higher to 3-fold-higher risk of cardiovascular (CV) mortality compared with those without DM, and the Da Qing IGT and Diabetes Study reported that newly diagnosed DM increases CV mortality by 2.7-fold in men and by 5.9-fold in women during 23 years of follow-up.^[2–4]^

Alteration in CV sympathetic innervation may influence the heart rate-corrected QT interval (QTc interval), and QTc prolongation may predispose patients to arrhythmia and sudden CV death.^[5]^ It is reported that the QTc interval is associated with all-cause mortality or CV mortality in the general population, and several studies have reported this association among patients with T2DM, which was evaluated by a simple, reproducible method.^[6–8]^

Cardiovascular autonomic neuropathy (CAN) is associated with increased all-cause mortality regardless of traditional CV risk factors, but it is mainly an underdiagnosed complication of T2DM.^[9]^ CAN is the consequential damage to the autonomic nerve fibers of the CV system, resulting in abnormalities in the heart rate control and vascular dynamics.^[10]^ The prevalence of CAN in T2DM patients was reported as 31% to 73%, according to the study participants’ diagnostic criteria, test method, age, and duration of DM.^[10]^

Because CAN frequently remains in a subclinical state before progressing to an advanced state, it would be helpful to predict CAN in the early stages.^[9]^ Standardized CV reflex testing, including the RR response to deep breathing, Valsalva maneuver, and postural changes in blood pressure, is the gold standard for the diagnosis of CAN by the Toronto Consensus and neurological scientific societies.^[11]^ Decreased beat-to-beat variation, which can be assessed by evaluating heart rate variability (HRV), might be one of the easiest and most reliable ways to assess CAN in the early stages.^[9,12]^

Pappachan et al^[13]^ reported the association of the standardized CV reflex with the QTc interval in T2DM patients; however, the association between the QTc interval and CAN, especially HRV in T2DM, is still unclear. Further, these tests for the diagnosis of CAN can be cumbersome to perform in primary care clinical settings. Therefore, this study aimed to estimate the association between HRV and a prolonged QTc interval in patients with T2DM to understand the increased CV risk in T2DM better.

## 2. Materials and methods

### 2.1. Study participants

The cross-sectional study recruited 411 patients aged 25 to 69 years in succession and performed an autonomic function test (AFT) and resting 12-lead electrocardiography (ECG) in the patients between March 1, 2009, and December 31, 2009.

Patients with T1DM, gestational DM, liver cirrhosis, end-stage renal disease, malignancy, sepsis, a history of coronary heart disease or cerebrovascular disease, a history of arrhythmia (including atrial fibrillation and bundle-branch block, Wolff–Parkinson–White syndrome, and use of a pacemaker) or a baseline ECG result with arrhythmia, and patients taking medications that could influence the AFT result or QTc interval measurement were excluded. All participants provided written consent to participate in the study, and the ethics committee of Catholic Medical Center approved the research protocol.

At the time of enrollment, all participants were interviewed regarding their medical history and subjected to detailed anthropometric investigations, as described in previous publications.^[14]^ Hypertension was defined as a systolic blood pressure of ≥ 140 mm Hg, a diastolic blood pressure of ≥ 90 mm Hg, or the use of antihypertensive drugs. Tobacco use was defined as current cigarette smoking. Body mass index was defined as the body weight (kg) divided by the square of the height (m).

Laboratory variables, such as the fasting plasma glucose level and lipid profile, were measured using automated enzymatic methods (736–40; Hitachi, Tokyo, Japan) after fasting for 8 hours, and hemoglobin A1c (HbA1c) was measured by high-performance liquid chromatography (Montreal, Canada). Diabetic nephropathy was defined as a urine albumin-to-creatinine ratio of > 30 mg/g creatinine in spot-urine specimens, which was confirmed at least twice in 6 months.^[15]^

### 2.2. Cardiovascular autonomic function test and 12-lead electrocardiography

All participants were instructed to avoid alcohol, nicotine, and caffeine and fast for 8 hours prior to undergoing the AFT. After a 10-minute break, the participants were subjected to HRV measurements using DiCAN (Medicore, Seoul, Korea) between 8 am and 12 pm to avoid changes in drivability. The participants sat on chairs in a comfortable position, and electrodes were attached to their wrists and left feet for 5 minutes to determine the HRV.^[16]^

The variables measuring HRV were recorded for 5 minutes by time- and frequency-domain analysis, and variations, such as the standard deviation of the normal-to-normal interval (SDNN), root-mean-square of successive differences between adjacent RR intervals (RMSSD), total power (TP), low frequency (LF) (0.04–0.15), and high frequency (HF) (0.15–0.40 Hz), could be accurately explained. TP is the variance of the normal-to-normal interval over the time segment.^[17]^

To compare variables measuring HRV, we also performed the CV reflex test using Ewing’s method.^[18]^ The expiratory/inspiratory difference was calculated as the average difference in the expiration and inhalation of the RR interval over six consecutive breaths during inhalation at a rate of 6 breaths per minute. The ratio of 30:15 is the ratio of the longest RR interval of 20 to 40 beats after standing to the shortest RR interval of 5 to 25 beats after standing. The Valsalva maneuver is the ratio of 40 mm Hg for 15 s to the longest RR interval during forced exhalation in the mouthpiece of the pressure gauge.^[18]^ An expiratory/inspiratory difference of ≤ 10, Valsalva ratio of ≤ 1.10, and posture ratio of ≤ 1.00 were considered abnormal. Each measurement was scored as normal (0) or abnormal (1), and the total score was calculated as the sum of the CAN scores. The CAN stage was defined as follows: A score of 0 was defined as normal, a score of 1 was defined as an early stage of CAN, and a score of at least 2 was defined as a definite diagnosis of CAN.^[19,20]^

Digital 12-lead ECGs were recorded on the same day using an ECG device (PI-19E; Tenshi Fukuda, Tokyo, Japan). The ECG recorded all leads simultaneously, facilitating an accurate evaluation of the QT interval, defined as the interval in the cardiac electrical cycle from the beginning of the QRS complex to the end of the T wave. The QT and RR intervals were measured using five consecutive beats of lead II by two experienced physicians blinded to the diagnosis. The QT interval was corrected for the heart rate using Bazett’s formula (QTc interval = QT/RR^1/2^) and a linear regression-based formula as calculated by Rautaharju et al.^[21]^ QTc measurements of > 440 ms were considered abnormally prolonged.^[22]^ The variability between observers in the QT interval, which was evaluated using the coefficient of variation, was 3.1%.^[23]^

### 2.3. Statistical analysis

Continuous variables were reported as the mean ± standard deviation or as the median (interquartile range) and were compared using Student’s *t* test for continuous symmetric variables and the Mann–Whitney *U* test for continuous asymmetric variables. Dichotomic variables were described as frequencies and percentages and were compared using the chi-square test. Logistic regression analysis was performed to assess the relationship between the quartiles of the time-domain parameters or frequency-domain parameters of the variables measuring HRV and QTc interval prolongation. Multiple linear regression analysis was performed to assess the relationship between the variables measuring HRV and the QTc interval. Explanatory variables were checked for confounding factors, and multicollinearity was checked if the variance inflation factor was > 10. Statistical significance was evaluated with a significance level of 0.05 for both tests. The statistical analysis was performed using IBM SPSS Statistics for Windows, version 26.0 (IBM Corp., Armonk, NY).

## 3. Results

Of the 507 participants screened, 96 participants were excluded, including 16 patients with T1DM, 4 patients with end-stage renal disease, 3 patients with liver cirrhosis, 4 patients with atrial fibrillation, 51 patients who were taking drugs influencing AFT, and 18 patients with malignancy. After applying the exclusion criteria, 411 patients with T2DM were enrolled in this study.

The baseline characteristics of the study participants are presented in Table 1. The median baseline QTc interval in this study was 423 ms [range: 365–570 ms], and the interquartile range was 407 to 438.

**Table 1:** Baseline characteristics of the patients with type 2 diabetes included in this study according to QT interval prolongation.

	Total (n = 411)	QT prolongation (−) (n = 321)	QT prolongation (+) (n = 90)	*P* value
Age (yr)	56.3 ± 10.6	55.5 ± 10.6	59.4 ± 10.1	.002
Sex (Female, %)	231 (56.2)	166 (51.7)	65 (72.2)	.001
Diabetic duration (yr)	9.6 ± 7.3	9.2 ± 7.2	10.9 ± 7.7	.064
BMI (kg/m^2^)	24.7 ± 3.6	24.8 ± 3.5	24.5 ± 3.9	.466
Smoking (current)	83 (21.8)	69 (23.3)	14 (16.5)	.331
Hypertension (%)	270 (34.7)	235 (33.4)	35 (46.7)	.022
SBP (mm Hg)	123.4 ± 17.4	121.8 ± 15.1	129.0 ± 23.2	<.001
DBP (mm Hg)	80.4 ± 10.2	80.0 ± 9.8	81.8 ± 11.4	.133
Laboratory variables				
FPG (mg/dL)	158.2 ± 57.1	155.7 ± 56.6	166.9 ± 58.5	.112
pp2hr (mg/dL)	250.4 ± 81.2	243.9 ± 78.9	273.2 ± 85.7	.004
HbA1c (%)	8.4 ± 1.9	8.2 ± 1.8	8.8 ± 2.2	.045
Creatinine (mg/dL)	0.8 ± 0.2	0.8 ± 0.2	0.8 ± 0.3	.149
Total cholesterol (mg/dL)	185.2 ± 39.4	186.7 ± 37.8	179.6 ± 44.5	.178
Triglyceride (mg/dL)	132.2 ± 79.7	133.4 ± 77.4	127.9 ± 87.8	.573
HDL-C (mg/dL)	44.3 ± 10.0	44.2 ± 10.3	44.6 ± 8.9	.745
LDL-C (mg/dL)	114.4 ± 34.9	115.8 ± 33.9	109.4 ± 37.9	.135
C-peptide (ng/mL)	1.7 ± 1.2	1.7 ± 1.2	1.5 ± 0.9	.288
ACR (mg/gCr)	113.9 (74.7–171.9)	118.2 (77.9–179.0)	98.7 (58.9–135.4)	.212
Medication (%)				
Insulin	110 (26.8)	72 (22.4)	38 (42.2)	<.001
Sulfonylurea	250 (60.8)	194 (60.4)	56 (62.2)	.759
Metformin	271 (65.9)	213 (66.4)	58 (64.4)	.735
ACEi/ARB	126 (30.7)	97 (30.2)	29 (32.2)	.716
Beta blocker	15 (3.6)	13 (4.0)	2 (2.2)	.414
CCB	66 (16.1)	47 (14.6)	19 (21.1)	.140
Aspirin	64 (15.6)	49 (15.3)	15 (16.7)	.746
Statin	115 (28.0)	91 (28.4)	24 (26.7)	.741
Mean HR (beats/min)	73.5 (67.8–83.2)	72.4 (66.7–79.7)	81.0 (72.7–89.0)	<.001
Time-domain parameters				
SDNN (milliseconds)	24.8 (17.7–33.1)	27.2 (19.7–34.4)	18.2 (12.6–28.0)	<.001
RMSSD (milliseconds)	14.2 (9.4–21.7)	14.9 (10.5–22.9)	10.9 (7.3–16.8)	<.001
Frequency-domain parameters				
TP (milliseconds^2^)	291.3 (142.6–637.6)	328.7 (185.7–721.7)	147.7 (74.1–335.9)	.002
HF (milliseconds^2^)	43.1 (18.3–107.2)	51.6 (22.4–122.1)	19.6 (7.2–53.1)	<.001
LF (milliseconds^2^)	60.6 (23.6–150.3)	67.9 (33.8–182.2)	23.6 (12.6–64.9)	.005
LF/HF ratio	1.4 (0.7–2.9)	1.4 (0.7–2.9)	1.5 (0.6–2.7)	.371
QT interval (milliseconds)				
QT interval (Bazett)	423 (407–438)	417 (403–428)	450 (446–458)	<.001
QT interval (Linear regression function)	417 (402–428)	410 (399–420)	437 (431–445)	<.001

Data are number (percentage), means ± SD, or median (interquartile range). *P* < .05 was considered significant.

ACEi/ARB = ACE inhibitor/angiotensin receptor blocker, ACR = albumin-to-creatinine ratio, BMI = body mass index, CCB = calcium channel blocker, DBP = diastolic blood pressure, FPG = fasting plasma glucose, HbA1c = glycated hemoglobin A1c, HDL = high-density lipoprotein, HF = high-frequency, HR = heart rate, LDL = low-density lipoprotein, LF = low-frequency, pp2hr = postprandial 2 hr glucose, QT interval (Bazett) = heart rate–corrected QT interval by Bazett formula, QT interval (Linear regression function) = heart rate-corrected QT interval by Rautaharju and Zhang, RMSSD = square root of the mean squared difference of successive RR intervals, SBP = systolic blood pressure, SDNN = standard deviation of normal RR intervals, TP = total power.

The study participants consisted of 231 (56.2%) female participants. At baseline, the study participants had a mean age and DM duration of 56.3 ± 10.6 years and 9.6 ± 7.3 years, respectively. A total of 90 patients in this study had QTc interval prolongation (21.9%).

The participants with a prolonged QTc interval were older (59.4 ± 10.1 years vs 55.5 ± 10.6 years, *P* = .002), were more likely to be a woman (72.2% vs 51.7%, *P* = .001), had a higher prevalence of hypertension (46.7% vs 33.4%, *P* = .022), had a higher systolic blood pressure (129.0 ± 23.2 mm Hg vs 121.8 ± 15.1 mm Hg, *P* < .001), had a higher 2-hour postprandial glucose level (273.2 ± 85.7 vs 243.9 ± 78.9 mg/dL, *P* = .004), had a higher HbA1c level (8.8% ± 2.2% vs 8.2% ± 1.8%, *P* = .045), and had a higher rate of insulin use (42.2% vs 22.4%, *P* < .001). Moreover, the participants with a prolonged QTc interval had decreased HRV parameters, including time- and frequency-domain parameters, except for the LF/HF ratio (Table 1). While the HRV parameters differed according to QTc interval prolongation, the CAN stage did not differ according to QTc interval prolongation (*P* = .854) (see Table S1, Supplemental Digital Content, http://links.lww.com/MD/H813, which illustrates baseline CAN stage according to QTc interval prolongation in patients with type 2 diabetes).

In the estimations of the QTc interval and HRV parameters according to sex, male and female patients with a prolonged QTc interval exhibited decreased HRV parameters, except for the LF/HF ratio (see Table S2, Supplemental Digital Content, http://links.lww.com/MD/H814, which illustrates HRV variables according to QTc interval prolongation stratified by sex).

**Figure 1.**
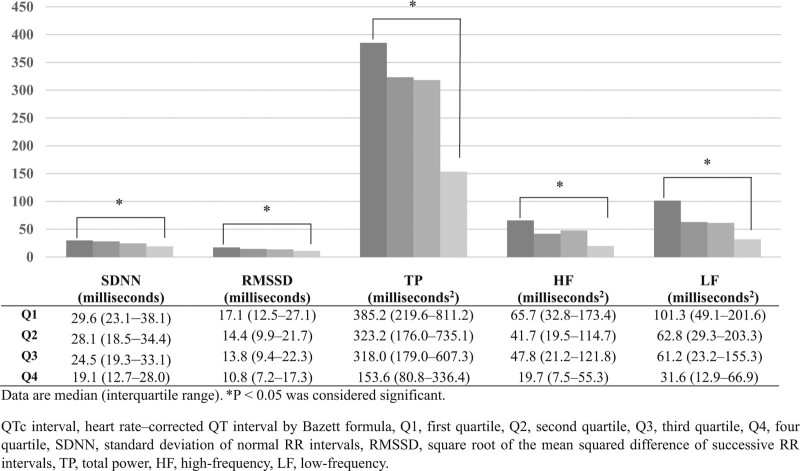
Time-and frequency-domain measures of heart rate variability according to the quartile of heart rate-corrected QT interval in patients with type 2 diabetes. Data are median (interquartile range). *P* < .05 was considered significant. HF = high-frequency, LF = low-frequency, Q1 = first quartile, Q2 = second quartile, Q3 = third quartile, Q4 = four quartile, QTc interval = heart rate–corrected QT interval by Bazett formula, RMSSD = square root of the mean squared difference of successive RR intervals, SDNN = standard deviation of normal RR intervals, TP = total power.

We then divided the patients into four groups according to the QTc interval measurement. Regarding the quartiles of the QTc interval, patients with a longer QTc interval tended to exhibit a low HRV, including for the SDNN, RMSSD, TP, HF, and LF variables (*P* for trend < 0.05), except for the LF/HF ratio (Fig. 1).

Figure 1 shows the relationship between QTc interval prolongation and HRV variables in T2DM patients. After adjusting for age, sex, DM duration, baseline HbA1c level, and presence of diabetic nephropathy, logistic regression analysis demonstrated that QTc interval prolongation was associated with the lowest quartiles of the HRV parameters including SDNN (odds ratio [OR] = 3.31; 95% CI: 1.89–5.77), RMSSD (OR = 1.98; 95% CI: 1.13–3.47), TP (OR = 3.99; 95% CI: 2.29–6.96), HF (OR = 3.20; 95% CI: 1.84–5.58), and LF (OR = 3.68; 95% CI: 2.10–6.43) in patients with T2DM (Table 2 and Table S3, Supplemental Digital Content, http://links.lww.com/MD/H815, which illustrates multiple logistic regression analysis of QTc interval prolongation in patients with type 2 diabetes).

**Table 2:** Multiple logistic regression analysis of heart rate-corrected QT interval prolongation in terms of heart rate variability and the pulse wave velocity in patients with type 2 diabetes.

	Odds ratio	95% CI	*P* value
Time-domain parameters			
Lowest quartile of SDNN	3.306	1.894–5.771	<.001
Lowest quartile of RMSSD	1.980	1.129–3.470	.017
Frequency-domain parameters			
Lowest quartile of TP	3.993	2.291–6.958	<.001
Lowest quartile of HF	3.200	1.835–5.582	<.001
Lowest quartile of LF	3.675	2.102–6.426	<.001
Lowest quartile of LF/HF ratio	1.372	0.777–2.423	.276

Heart rate-corrected QT interval (QTc) prolongation, QTc > 440 ms.

HF = high-frequency, LF = low-frequency, RMSSD = square root of the mean squared difference of successive RR intervals, SDNN = standard deviation of normal RR intervals, TP = total power.

Multiple regression analysis was also performed using the QTc interval as the dependent variable and age, sex, HbA1c level, DM duration, and variables measuring HRV as independent variables. After adjusting for age, sex, DM duration, and baseline HbA1c level, the SDNN, RMSSD, TP, LF, and HF predicted the QTc interval (see Table S4, Supplemental Digital Content, http://links.lww.com/MD/H816, which illustrates multiple linear regression analysis of QTc interval prolongation with HRV variable). Except for the variables measuring HRV, the QTc interval was positively correlated with age, sex, and HbA1c level.

## 4. Discussion

In this study, we demonstrated that the prolongation of the QTc interval, measured using standard resting 12-lead ECG, is associated with decreased values for the variables measuring HRV in patients with T2DM, while the CAN stage is not associated with this. In addition, the association between the QTc interval prolongation and HRV parameters was significant, regardless of age, sex, DM duration, HbA1c level, and presence of diabetic nephropathy.

The clinical importance of QTc interval prolongation in patients with T2DM is emphasized with the further risk of arrhythmia and increased all-cause mortality, although the mechanism for this is not clearly understood. In this study, the prevalence of QTc interval prolongation in patients with T2DM was 21.9%, which is slightly lower than the prevalence reported by a previous observation by Li X et al, which other studies also exhibited diversity according to the definition of QT interval prolongation, heart rate correction method, or participants’ age, sex ratio, DM duration, and use of insulin. This study excluded those with previous CV disease and a history of arrhythmia, as well as participants taking medications that could influence the QTc interval; thus, the prevalence of QTc interval prolongation in this study may be underestimated compared with that in other studies.^[24–26]^

The EURODIAB IDDM Complications study demonstrated that a QT interval of > 440 ms was associated with impaired HRV in male patients with T1DM.^[27]^ It is reported that the relationship between the QTc interval and CAN in T2DM is unclear.^[19]^ Takahashi et al reported that baroreflex sensitivity was negatively correlated with the QTc interval, whereas the HF power and LF/HF ratio were not.^[28]^ The HF power and baroreflex sensitivity are both indexes of vagal activity. The HF power reflects the tonic vagal activity, whereas the baroreflex sensitivity reflects the reflex vagal activity. Additionally, only 42 Japanese patients with T2DM were included in this study.^[28]^ In addition to the QT interval, the QT distance might be used as an index for diagnosing CAN, and the QT distance is increased in patients with sensory neuropathy.^[29–31]^ QT interval variability is also considered a severity index for CAN.^[32]^ This study suggests that decreased HRV is associated with the QTc interval, which could be performed as a 12-lead resting ECG in a simple and noninvasive manner and could be assessed in primary care clinical practice for patients with T2DM.

CAN is usually subclinical in patients with T2DM. The association between CAN and all-cause mortality and CV events has been previously reported.^[9,33]^ Consequently, the early diagnosis of CAN is important. In the advanced stage, the clinical symptoms of CAN, including resting tachycardia and orthostatic hypotension, may develop, but HRV assessments may be necessary to detect asymptomatic CAN at the early stage.^[34]^ HRV can be evaluated using a time-domain measure by simply calculating the difference between the longest and shortest RR intervals. The SDNN is the square root of the variance, which is mathematically equal to the TP of the spectral analysis. The RMSSD is the square root of the mean-squared difference of successive RR intervals. Frequency domain methods provide basic information on the power distribution as a function of frequency. Two or three main spectral components, LF, HF, or very LF, LF, and HF are not fixed and may vary in changes in autonomic modulation of the heart cycle.^[17]^ Vagal activity is the major contributor to the HF component, whereas LF is a marker of sympathetic modulation.^[17]^

The mechanism of CAN is not fully understood but is known to involve multiple etiologies including hyperglycemia, intrinsic neuronal injury, or malperfusion in T2DM or T1DM.^[35]^ Hyperglycemia activates the polyol pathway, resulting in direct neural damage or dysfunction and reduced neuronal blood flow.^[35,36]^ Protein kinase C activation has been reported to reduce neuronal blood flow, subsequently increasing reactive oxygen species, which leads to endothelial damage, gene activation, and energy deficit with neuronal damage.^[37–39]^ Low-grade inflammation mediated by nuclear factor kappa B (NF-κB) activation has been found to play an important role in the pathogenesis of diabetic neuropathy with deficits in peripheral and autonomic nerve fibers.^[40]^

Moreover, the Action to Control Cardiovascular Risk in Diabetes trial demonstrated that, in those in the lowest quartile of the SDNN, a prolonged QT interval was independently associated with an increased risk of CV mortality by 1.9-fold and increased risk of all-cause mortality by 1.6-fold in participants with T2DM, indicating that reduced HRV and the coexistence of a prolonged QTc interval could increase the all-cause mortality or CV mortality.^[41]^

The potential mechanism associated with QTc interval prolongation and decreased HRV also has not been clarified. Several mechanisms of QTc interval prolongation have been reported, including an imbalance of the CV autonomic system, gene mutations affecting cardiac ion channels involved in cardiac repolarization, post-myocardial infarction scar tissue, hyperglycemia or hypoglycemia, hyperinsulinemia, obesity, and ventricular hypertrophy.^[42–48]^

Additionally, the patients with T2DM with QTc interval prolongation in this study were older, had higher blood pressure, and had higher 2-hour postprandial and HbA1c levels, so these may be additional CV risk factors. Kobayashi et al exhibited that QTc interval prolongation occurred with the multiplicity of microvascular complications, which might be a result of a small infarction in the myocardium or early atherosclerosis. Although we excluded those with a history of CV disease, subclinical cardiac disease could be a risk factor for decreased HRV.^[49]^

A limitation of this study is that it was a cross-sectional study with a relatively small number of participants. Future prospective studies are needed to estimate the causal relationship between QTc prolongation and HRV. Second, this study had no data on the prevalence of death or future CV disease.

In conclusion, decreased HRV was associated with QTc interval prolongation in patients with T2DM, which suggests a possible relationship between a reduced abnormal autonomic function and QTc interval. Therefore, we recommend that the QTc interval measurement may be valuable in the screening and follow-up of patients with T2DM in clinical practice.

## Author contributions

**Conceptualization:** Seon-Ah Cha.

**Data curation**: Seon-Ah Cha.

**Formal analysis:** Seon-Ah Cha.

**Investigation:** Seon-Ah Cha.

**Methodology:** Seon-Ah Cha.

**Project administration:** Seon-Ah Cha.

**Supervision:** Seon-Ah Cha.

**Validation:** Seon-Ah Cha.

**Visualization:** Seon-Ah Cha.

**Writing – original draft:** Seon-Ah Cha.

**Writing – review & editing:** Seon-Ah Cha.

## Supplementary Material



## References

[1] Dianna J. Magliano, Edward J. Boyko IDF. Diabetes Atlas. 10th ed. Brussels, Belgium: Scientific Committee, 2021.

[2] Raghavan S Vassy JL Ho YL. Diabetes mellitus-related all-cause and cardiovascular mortality in a national cohort of adults. J Am Heart Assoc. 2019;8:e011295. 30776949 10.1161/JAHA.118.011295 PMC6405678

[3] Li G Zhang P Wang J. Cardiovascular mortality, all-cause mortality, and diabetes incidence after lifestyle intervention for people with impaired glucose tolerance in the Da Qing Diabetes Prevention Study: a 23-year follow-up study. Lancet Diabetes Endocrinol. 2014;2:474–80. 24731674 10.1016/S2213-8587(14)70057-9

[4] Stamler J Vaccaro O Neaton JD. Diabetes, other risk factors, and 12-yr cardiovascular mortality for men screened in the Multiple Risk Factor Intervention Trial. Diabetes Care. 1993;16:434–44. 8432214 10.2337/diacare.16.2.434

[5] Okin PM Devereux RB Howard BV. Assessment of QT interval and QT dispersion for prediction of all-cause and cardiovascular mortality in American Indians: the Strong Heart Study. Circulation. 2000;101:61–6. 10618305 10.1161/01.cir.101.1.61

[6] Noseworthy PA Peloso GM Hwang SJ. QT interval and long-term mortality risk in the Framingham Heart Study. Ann Noninvasive Electrocardiol. 2012;17:340–8. 23094880 10.1111/j.1542-474X.2012.00535.x PMC3481183

[7] Cox AJ Azeem A Yeboah J. Heart rate-corrected QT interval is an independent predictor of all-cause and cardiovascular mortality in individuals with type 2 diabetes: the Diabetes Heart Study. Diabetes Care. 2014;37:1454–61. 24574343 10.2337/dc13-1257 PMC4182905

[8] Wang S He Y Xu L. Association between QTc interval prolongation and outcomes of diabetic foot ulcers: data from a 4-year follow-up study in China. Diabetes Res Clin Pract. 2018;138:26–34. 29382590 10.1016/j.diabres.2018.01.021

[9] Draznin B Aroda VR Bakris G. 12. Retinopathy, Neuropathy, and Foot Care: Standards of Medical Care in Diabetes-2022. Diabetes Care. 2022;45(Suppl 1):S185–94. 34964887 10.2337/dc22-S012

[10] Fisher VL Tahrani AA. Cardiac autonomic neuropathy in patients with diabetes mellitus: current perspectives. Diabetes Metab Syndr Obes. 2017;10:419–34. 29062239 10.2147/DMSO.S129797 PMC5638575

[11] Spallone V. Update on the impact, diagnosis and management of cardiovascular autonomic neuropathy in diabetes: what is defined, what is new, and what is unmet. Diabetes Metab J. 2019;43:3–30. 30793549 10.4093/dmj.2018.0259 PMC6387879

[12] Shaffer F Ginsberg JP. An overview of heart rate variability metrics and norms. Front Public Health. 2017;5:258. 29034226 10.3389/fpubh.2017.00258 PMC5624990

[13] Pappachan JM Sebastian J Bino BC. Cardiac autonomic neuropathy in diabetes mellitus: prevalence, risk factors and utility of corrected QT interval in the ECG for its diagnosis. Postgrad Med J. 2008;84:205–10. 18424578 10.1136/pgmj.2007.064048

[14] Cha SA Yun JS Lim TS. Diabetic cardiovascular autonomic neuropathy predicts recurrent cardiovascular diseases in patients with type 2 diabetes. PLoS One. 2016;11:e0164807e0164807. 27741306 10.1371/journal.pone.0164807 PMC5065186

[15] Consensus development conference on the diagnosis and management of nephropathy in patients with diabetes mellitus. American Diabetes Association and the National Kidney Foundation. Diabetes Care. 1994;17:1357–61. 7821182 10.2337/diacare.17.11.1357

[16] Cha SA Park YM Yun JS. Time- and frequency-domain measures of heart rate variability predict cardiovascular outcome in patients with type 2 diabetes. Diabetes Res Clin Pract. 2018;143:159–69. 30006307 10.1016/j.diabres.2018.07.001 PMC6278593

[17] Heart rate variability: standards of measurement, physiological interpretation and clinical use. Task Force of the European Society of Cardiology and the North American Society of Pacing and Electrophysiology. Circulation. 1996;93:1043–65. 8598068

[18] Ewing DJ Martyn CN Young RJ. The value of cardiovascular autonomic function tests: 10 years experience in diabetes. Diabetes Care. 1985;8:491–8. 4053936 10.2337/diacare.8.5.491

[19] Spallone V Ziegler D Freeman R. Cardiovascular autonomic neuropathy in diabetes: clinical impact, assessment, diagnosis, and management. Diabetes Metab Res Rev. 2011;27:639–53. 21695768 10.1002/dmrr.1239

[20] Tesfaye S Boulton AJ Dyck PJ. Diabetic neuropathies: update on definitions, diagnostic criteria, estimation of severity, and treatments. Diabetes Care. 2010;33:2285–93. 20876709 10.2337/dc10-1303 PMC2945176

[21] Rautaharju PM Zhang ZM Prineas R. Assessment of prolonged QT and JT intervals in ventricular conduction defects. Am J Cardiol. 2004;93:1017–21. 15081446 10.1016/j.amjcard.2003.12.055

[22] Whitsel EA Boyko EJ Siscovick DS. Reassessing the role of QTc in the diagnosis of autonomic failure among patients with diabetes: a meta-analysis. Diabetes Care. 2000;23:241–7. 10868838 10.2337/diacare.23.2.241

[23] Cha SA Yun JS Lim TS. Baseline-Corrected QT (QTc) interval is associated with prolongation of QTc during severe hypoglycemia in patients with type 2 diabetes mellitus. Diabetes Metab J. 2016;40:463–72. 27766792 10.4093/dmj.2016.40.6.463 PMC5167711

[24] Cardoso CR Salles GF Deccache W. Prognostic value of QT interval parameters in type 2 diabetes mellitus: results of a long-term follow-up prospective study. J Diabetes Complications. 2003;17:169–78. 12810239 10.1016/s1056-8727(02)00206-4

[25] Hashimoto Y Tanaka M Senmaru T. Heart rate-corrected QT interval is a novel risk marker for the progression of albuminuria in people with Type 2 diabetes. Diabet Med. 2015;32:1221–6. 25683576 10.1111/dme.12728

[26] Li X Ren H Xu ZR. Prevalence and risk factors of prolonged QTc interval among Chinese patients with type 2 diabetes. Exp Diabetes Res. 2012;2012:234084. 23319939 10.1155/2012/234084 PMC3540769

[27] Veglio M Borra M Stevens LK. The relation between QTc interval prolongation and diabetic complications. The EURODIAB IDDM Complication Study Group. Diabetologia. 1999;42:68–75. 10027581 10.1007/s001250051115

[28] Takahashi N Nakagawa M Saikawa T. Regulation of QT indices mediated by autonomic nervous function in patients with type 2 diabetes. Int J Cardiol. 2004;96:375–9. 15301890 10.1016/j.ijcard.2003.07.026

[29] Vasheghani M Sarvghadi F Beyranvand MR. The relationship between QT interval indices with cardiac autonomic neuropathy in diabetic patients: a case control study. Diabetol Metab Syndr. 2020;12:102. 33292470 10.1186/s13098-020-00609-0 PMC7678155

[30] Statsenko ME Turkina SV Shalaeva SS. [Impaired cardiac structural and functional parameters in patients with chronic heart failure and diabetic cardiac autonomic neuropathy]. Ter Arkh. 2013;85:23–8. 24437214

[31] Tanikawa T Abe H Tanaka Y. Cardiac autonomic balance and QT dispersion during head-up tilt testing in diabetics with and without sensory neuropathy. Clin Exp Hypertens. 2004;26:137–44. 15038624 10.1081/ceh-120028551

[32] Khandoker AH Imam MH Couderc JP. QT variability index changes with severity of cardiovascular autonomic neuropathy. IEEE Trans Inf Technol Biomed. 2012;16:900–6. 22929462 10.1109/TITB.2012.2205010

[33] Chyun DA Wackers FJ Inzucchi SE. Autonomic dysfunction independently predicts poor cardiovascular outcomes in asymptomatic individuals with type 2 diabetes in the DIAD study. SAGE Open Med. 2015;3:2050312114568476. 26770763 10.1177/2050312114568476 PMC4679226

[34] Vinik AI Casellini C Parson HK. Cardiac autonomic neuropathy in diabetes: a predictor of cardiometabolic events. Front Neurosci. 2018;12:591. 30210276 10.3389/fnins.2018.00591 PMC6119724

[35] Pop-Busui R. Cardiac autonomic neuropathy in diabetes: a clinical perspective. Diabetes Care. 2010;33:434–41. 20103559 10.2337/dc09-1294 PMC2809298

[36] Vallianou N Evangelopoulos A Koutalas P. Alpha-lipoic acid and diabetic neuropathy. Rev Diabet Stud. 2009;6:230–6. 20043035 10.1900/RDS.2009.6.230 PMC2836194

[37] Doupis J Lyons TE Wu S. Microvascular reactivity and inflammatory cytokines in painful and painless peripheral diabetic neuropathy. J Clin Endocrinol Metab. 2009;94:2157–63. 19276232 10.1210/jc.2008-2385 PMC2690431

[38] Obrosova IG. How does glucose generate oxidative stress in peripheral nerve? Int Rev Neurobiol. 2002;50:3–35. 12198815 10.1016/s0074-7742(02)50071-4

[39] Pacher P Liaudet L Soriano FG. The role of poly(ADP-ribose) polymerase activation in the development of myocardial and endothelial dysfunction in diabetes. Diabetes. 2002;51:514–21. 11812763 10.2337/diabetes.51.2.514

[40] Wang Y Schmeichel AM Iida H. Enhanced inflammatory response via activation of NF-kappaB in acute experimental diabetic neuropathy subjected to ischemia-reperfusion injury. J Neurol Sci. 2006;247:47–52. 16631800 10.1016/j.jns.2006.03.011

[41] Pop-Busui R Evans GW Gerstein HC. Effects of cardiac autonomic dysfunction on mortality risk in the Action to Control Cardiovascular Risk in Diabetes (ACCORD) trial. Diabetes Care. 2010;33:1578–84. 20215456 10.2337/dc10-0125 PMC2890362

[42] Roden DM Spooner PM. Inherited long QT syndromes: a paradigm for understanding arrhythmogenesis. J Cardiovasc Electrophysiol. 1999;10:1664–83. 10636197 10.1111/j.1540-8167.1999.tb00231.x

[43] Peters RW Byington RP Barker A. Prognostic value of prolonged ventricular repolarization following myocardial infarction: the BHAT experience. The BHAT Study Group. J Clin Epidemiol. 1990;43:167–72. 2406377 10.1016/0895-4356(90)90180-w

[44] Marfella R Nappo F De Angelis L. The effect of acute hyperglycaemia on QTc duration in healthy man. Diabetologia. 2000;43:571–5. 10855531 10.1007/s001250051345

[45] Dekker JM Feskens EJ Schouten EG. QTc duration is associated with levels of insulin and glucose intolerance. The Zutphen Elderly Study. Diabetes. 1996;45:376–80. 8593946 10.2337/diab.45.3.376

[46] Carella MJ Mantz SL Rovner DR. Obesity, adiposity, and lengthening of the QT interval: improvement after weight loss. Int J Obes Relat Metab Disord. 1996;20:938–42. 8910099

[47] Rautaharju PM Park LP Chaitman BR. The Novacode criteria for classification of ECG abnormalities and their clinically significant progression and regression. J Electrocardiol. 1998;31:157–87. 9682893

[48] Gastaldelli A Emdin M Conforti F. Insulin prolongs the QTc interval in humans. Am J Physiol Regul Integr Comp Physiol. 2000;279:R2022–5. 11080065 10.1152/ajpregu.2000.279.6.R2022

[49] Kobayashi S Nagao M Asai A. Severity and multiplicity of microvascular complications are associated with QT interval prolongation in patients with type 2 diabetes. J Diabetes Investig. 2018;9:946–51. 10.1111/jdi.12772 PMC6031516 29095573

