# Supplementary material for: Heart rate-corrected QT interval prolongation is associated with decreased heart rate variability in patients with type 2 diabetes
Source: Medicine (Baltimore). 2022 Nov 11;101(45):e31511. doi: 10.1097/MD.0000000000031511 (PMC9666134; doi:10.1097/MD.0000000000031511)
Supplement: Supplementary file 1 [file medi-101-e31511-s001.pdf]

Table S1. Baseline cardiovascular autonomic neuropathy stage according to QT interval prolongation in patients with type 2 diabetes

	Total (n=411)	QT prolongation (-) (n=321)	QT prolongation (+) (n=90)	*P* value
**CAN stage**				0.854
**Normal**	206 (50.1)	161 (50.2)	45 (50.0)	
**Early**	148 (36.0)	117 (36.4)	31 (34.4)	
**Definite**	57 (13.9)	43 (13.4)	14 (15.6)	

Data are number (percentage). $P < 0.05$ was considered significant.

CAN, cardiovascular autonomic neuropathy.
