# Supplementary material for: Heart rate-corrected QT interval prolongation is associated with decreased heart rate variability in patients with type 2 diabetes
Source: Medicine (Baltimore). 2022 Nov 11;101(45):e31511. doi: 10.1097/MD.0000000000031511 (PMC9666134; doi:10.1097/MD.0000000000031511)
Supplement: Supplementary file 2 [file medi-101-e31511-s002.pdf]

Table S2. Comparison of variables measuring heart rate variability between patients with a prolonged heart rate-corrected QT interval stratified by sex in patients with type 2 diabetes

	Men			Women	
	QT prolongation (-) (n=155)	QT prolongation (+) (n=25)	*P* value	QT prolongation (-) (n=166)	Q
Mean HR (beats/minute)	72.4 (65.8–79.4)	84.0 (71.2–90.9)	< 0.001	72.4 (66.9–81.1)	
SDNN (ms)	29.1 (23.0–37.3)	17.1 (13.2–27.7)	< 0.001	24.0 (18.3–32.9)	
RMSSD (ms)	15.1 (11.2–22.7)	10.3 (7.1–16.0)	0.001	14.6 (9.6–23.5)	
TP (ms^2)	394.3 (212.5–830.2)	131.2 (62.9–344.7)	< 0.001	279.6 (169.8–598.8)	1
HF (ms^2)	56.1 (27.3–121.8)	19.8 (5.7–49.6)	< 0.001	44.3 (20.1–122.8)	
LF (ms^2)	92.1 (45.9–215.7)	37.1 (13.5–79.3)	< 0.001	56.7 (23.7–134.0)	
LF/HF Ratio	1.8 (0.9–3.5)	1.6 (0.6–3.9)	0.927	1.0 (0.7–2.4)	
QT interval (ms)					
QT interval (Bazett)	409 (396–424)	448 (446–455)	< 0.001	422 (409–429)	
QT interval (Linear regression function)	404 (393–417)	436 (432–440)	< 0.001	414 (404–421)	

Data are number (percentage), means ± SD, or median (interquartile range). P < 0.05 was considered significant.

HR, heart rate, SDNN, standard deviation of normal RR intervals, RMSSD, square root of the mean squared difference of successive RR intervals, TP, total power, HF, high-frequency, LF, low-frequency, QT interval (Bazett), heart rate –corrected QT interval by Bazett formula, QT interval (Linear regression function), heart rate-corrected QT interval by Rautaharju and Zhang.
