# Supplementary material for: Heart rate-corrected QT interval prolongation is associated with decreased heart rate variability in patients with type 2 diabetes
Source: Medicine (Baltimore). 2022 Nov 11;101(45):e31511. doi: 10.1097/MD.0000000000031511 (PMC9666134; doi:10.1097/MD.0000000000031511)
Supplement: Supplementary file 3 [file medi-101-e31511-s003.pdf]

Table S3. Multiple logistic regression analysis of heart rate-corrected QT interval prolongation in patients with type 2 diabetes

	Odds ratio	95% CI	*P* value
**Age, year**	1.035	1.005–1.065	0.024
**Female sex**	1.987	1.114–3.544	0.020
**Diabetes duration, year**	0.982	0.944–1.022	0.383
**HbA1c, %**	1.135	0.983–1.311	0.084
**Diabetic nephropathy**	1.428	0.812–2.511	0.216
**Lowest quartile of total power**	3.993	2.291–6.958	< 0.001

Heart rate-corrected QT interval (QTc) prolongation, QTc > 440ms.
