# Supplementary material for: Heart rate-corrected QT interval prolongation is associated with decreased heart rate variability in patients with type 2 diabetes
Source: Medicine (Baltimore). 2022 Nov 11;101(45):e31511. doi: 10.1097/MD.0000000000031511 (PMC9666134; doi:10.1097/MD.0000000000031511)
Supplement: Supplementary file 4 [file medi-101-e31511-s004.pdf]

Table S4. Multiple linear regression analysis of the association of the heart rate-corrected QT interval with variables measuring heart rate variability

	**ß ± SE**	***p* value**
**Time-domain parameters**		
**SDNN***	-0.220 ± 0.002	< 0.001
**RMSSD***	-0.210 ± 0.002	< 0.001
**Frequency-domain parameters**		
**TP***	-0.218 ± 0.001	< 0.001
**HF***	-0.232 ± 0.001	< 0.001
**LF***	-0.254 ± 0.001	< 0.001
**LF/HF Ratio***	-0.018 ± 0.001	0.712

*Log-transformed values were used for analysis.

SDNN, standard deviation of normal RR intervals, RMSSD, square root of the mean squared difference of successive RR intervals, TP, total power, HF, high-frequency, LF, low-frequency.
